# Comparison between 3D SPACE FLAIR and 3D TSE FLAIR in Menière’s disease

**DOI:** 10.1007/s00234-022-02913-0

**Published:** 2022-02-12

**Authors:** Anja Bernaerts, Nick Janssen, Floris L. Wuyts, Cathérine Blaivie, Robby Vanspauwen, Joost van Dinther, Andrzej Zarowski, Erwin Offeciers, Filip Deckers, Jan W. Casselman, Bert De Foer

**Affiliations:** 1 Department of Radiology, GZA Hospitals Antwerp, Oosterveldlaan 24, 2610 Antwerp, Belgium; 2 Lab for Equilibrium Investigations and Aerospace, University of Antwerp, Universiteitsplein 1, 2610 Antwerp, Belgium; 3 European Institute for ORL-HNS, GZA Hospitals Antwerp, Oosterveldlaan 24, 2610 Antwerp, Belgium; 4 Department of Radiology, AZ Sint-Jan Hospital, Ruddershove 10, 8000 Bruges, Belgium

**Keywords:** Magnetic resonance imaging, Menière disease, Endolymphatic hydrops, Perilymph, Diagnosis

## Abstract

**Purpose:**

Heavily T2-weighted 3D FLAIR (hT_2_w-3D-FLAIR) sequence with constant flip angle (CFA) has been reported as being more sensitive to low concentrations of gadolinium (Gd) enabling endolymphatic hydrops (EH) visualization. The purpose of this study was to compare signal-to-noise (SNR) ratio, detection rate of EH, and increased perilymphatic enhancement (PE) as well as diagnostic accuracy in diagnosing definite Menière’s disease (MD), using 3D-SPACE FLAIR versus conventional 3D-TSE FLAIR.

**Methods:**

This retrospective study included 29 definite MD patients who underwent a 4-h delayed intravenous (IV) Gd-enhanced 3D-TSE FLAIR and 3D-SPACE FLAIR MRI between February 2019 and February 2020. MR images were qualitatively and quantitatively analyzed twice by 2 experienced head and neck radiologists. Qualitative assessment included grading of cochlear and vestibular EH and visual comparison of PE. Quantitative assessment of PE was performed by placing a region of interest (ROI) and ratio calculation in the basal turn of the cochlea and the brainstem.

**Results:**

The intra- and inter-reader reliability for grading of EH and PE was excellent (0.7 < kappa < 0.9) for 3D-SPACE FLAIR and exceeded the values for 3D-TSE FLAIR (0.5 < kappa < 0.9) The combination of EH and visual assessment of PE has the highest diagnostic accuracy in diagnosing definite MD on 3D-SPACE FLAIR with a sensitivity of 0.91 and a specificity of 0.98 resulting in a sensitivity raise of 6% compared to 3D-TSE FLAIR.

**Conclusion:**

Four-hour delayed IV Gd-enhanced 3D-SPACE FLAIR sequence has a higher sensitivity and reproducibility than 3D-TSE FLAIR for the visualization of EH and increased PE in definite MD patients.

## Introduction

In the past decade, it has become feasible to discriminate endolymphatic hydrops (EH)—the morphological substrate of Menière’s disease (MD)—using delayed post-gadolinium (Gd) magnetic resonance imaging (MRI) [1–6]. Combining additional image markers with EH such as the degree of perilymphatic enhancement (PE) has recently been described as highly specific for MD [3, 5]. The intravenous gadolinium (IV) administration has been widely adopted as the method of choice for hydrops imaging over the intratympanic (IT) method, with three-dimensional (3D) turbo spin-echo (TSE) fluid-attenuated inversion recovery (FLAIR) sequence as the sequence of choice over 3D REAL inversion recovery (IR) sequences, the former being more sensitive to T1-shortening [1, 2, 4, 6]. Whereas, originally, 3D REAL IR was a single sequence developed for the IT administration of Gd—in which by adapting the inversion time endolymphatic space, perilymphatic space, and surrounding bone can be separately visualized—it has nowadays evolved for IV Gd use into a process of subtraction of imaging series with a different inversion time and/or a heavily T2-weighted MR cisternography (MRC) sequence [7–10]. 3D TSE FLAIR does not require this complex post-processing involving subtraction of imaging series like 3D REAL IR sequences, being time-consuming, and with potential misregistration [2, 6].

Heavily T2-weighted 3D FLAIR (hT_2_w-3D-FLAIR) sequence with constant flip angle (CFA) has previously been reported in 2 small patient groups as being more sensitive to low concentrations of Gd enabling visualization of EH [11, 12]. 3D sampling perfection with application optimized contrasts using different flip angle evolution (SPACE) FLAIR is such a hT_2_w-3D-FLAIR and has a comparable spatial resolution to 3D TSE FLAIR. However, as compared to the 3D TSE FLAIR, 3D SPACE FLAIR has a higher radiofrequency (RF) receiver bandwidth with a constant (CFA) or variable flip angle (VFA), resulting in a higher echo train length and a longer echo time (TE) for the SPACE FLAIR. A CFA is to be preferred over a VFA because of the higher signal intensity ratio (SIR) [11]. The increased repetition time (TR) and TE give much more heavily T2-weighted images, which also benefits signal-to-noise ratio (SNR).

The purpose of this study was to compare in a larger group of patients with definite MD the SNR, the detection rate of EH and increased PE as well as its diagnostic accuracy in diagnosing MD, using 3D SPACE FLAIR with a CFA versus conventional 3D TSE FLAIR using a single dose of IV Gd.

## Materials and methods

### Patients

Between February 2019 and February 2020, 85 consecutive patients with Menièriform symptoms, such as tinnitus, vertigo, aural fullness, fluctuating hearing loss, and/or a combination of these symptoms, were referred for 3 T MRI of the temporal bone to demonstrate EH. All patients with a history of previous ear surgery or central disorders were excluded from the study. With institutional approval for the study (GZA study number: 200505 RETRO), the MRI data of these patients were retrospectively analyzed.

Patients were clinically evaluated by our ORL-HNS department and were diagnosed with probable or definite MD according to the 2015 Bárány Society criteria [13]. The classification of the clinical symptoms and functional tests was done by a vestibular clinical scientist (RVS with an experience of 13 years). Only patients classified as unilateral definite MD—29 in total—were included in this study. The contralateral normal ear was used as a control group.

### MRI protocol

MR examinations were performed on a 3 T Magnetom SkyraFit (Siemens Healthineers, Erlangen, Germany) with a 20-element head/neck coil. All patients underwent MR imaging 4 h after injection of a single dose of IV administrated Gd (Gadovist; Bayer- Schering Pharma, Berlin, Germany; 1.0 mmol/mL at a dose of 0.1 mmol/kg). The imaging protocol consisted of a conventional 3D TSE FLAIR and a 3D SPACE FLAIR with CFA. Detailed imaging parameters are listed in Table 1.

**Table 1:** Imaging parameters of 3D TSE FLAIR and 3D SPACE FLAIR

	3D TSE FLAIR	3D SPACE FLAIR
TR (ms)	5400	10,000
TE (ms)	146	549
TI (ms)	2000	2600
FOV (mm^2^)	150 × 160	160 × 160
Matrix	250 × 256	250 × 256
Number of slices	44	36
Acquired slice thickness (mm)	0.8	0.8
Averages	1	2
Parallel imaging	Grappa factor 2	Grappa factor 2
RF bandwidth (Hz/pixel)	271	501
Echo train length	26	235
Flip angle mode	Constant 180°	Constant 120°
Acquisition time (m:s)	12:38	07:10

Both sequences have a comparable spatial resolution. However, as compared to the 3D TSE with FLAIR contrast, activating the hyper-echo RF-scheme with CFA of 120° results in a higher echo train length and TE for the SPACE FLAIR. The increased TR and TE give much more heavily T2-weighted images. Moreover, the increased echo train length significantly reduces acquisition time, which benefits patient comfort and minimizes motion artefacts.

### Imaging analysis

MR images were qualitatively and quantitatively analyzed twice by 2 experienced head and neck radiologists (AB, and BDF, with experience in head and neck radiology of respectively 16 and 26 years) blinded to the scan parameters, clinical findings, and the side, uni-, or bilaterality of symptoms. All sequences were presented individually and randomized to both readers and not per patient in pairs. The time interval between the 2 readings was at least 4 weeks.

#### Qualitative assessment

Qualitative assessment included evaluation and grading of cochlear and vestibular EH and visual comparison of the PE.

We used the modified EH grading system according to Bernaerts et al. for cochlear hydrops (no hydrops, grade 1, 2) and vestibular hydrops (no hydrops, grade 1, 2, or 3) [3].

We also visually assessed the presence of cochlear blood-labyrinth barrier (BLB) impairment, defined as a subjective marked PE in the scala tympani of the basal turn of the cochlea relative to the contralateral cochlea, as also previously reported (less, equal, or more) in the paper of Bernaerts et al. [3].

#### Quantitative assessment

**Fig. 1:**
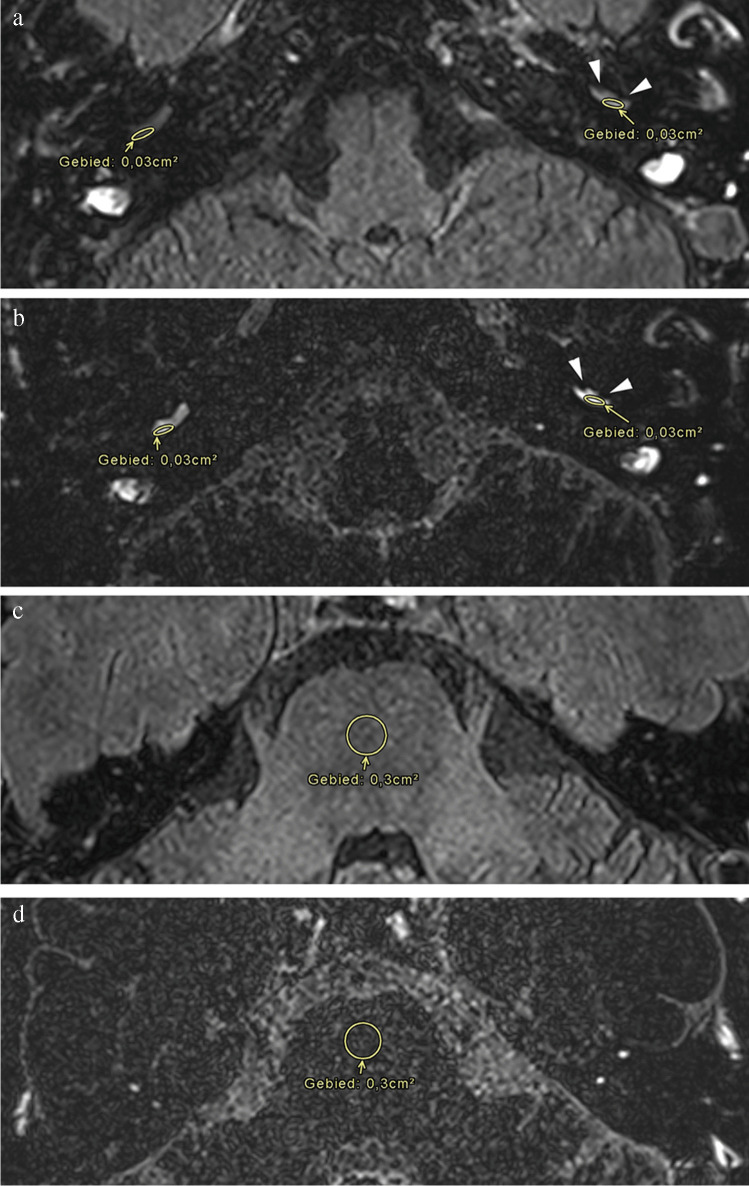
Forty-eight-year-old female with vertigo attacks, hearing loss on the left side associated with tinnitus, clinically classified as definite MD. Imaging performed 4 h after IV administration of a single dose of Gd. **a** Axial 3D TSE FLAIR image at the level of the basal turn of the cochlea. There is a visually asymmetrical perilymphatic enhancement more pronounced on the left side. A 3-mm^2^ elliptical ROI was placed in the scala tympani in the posterolateral part of the basal turn of the cochlea on both sides. **b** Axial 3D SPACE FLAIR image at the level of the basal turn of the cochlea. There is a visually asymmetrical perilymphatic enhancement more pronounced on the left side. Note the partial filling defect in the scala vestibuli of the basal turn of the cochlea on the left side by the enlarged endolympathic space (arrowheads) compatible with a cochlear EH grade I. This EH is hardly discernible on the 3D TSE FLAIR image (see Fig. 1a). A 3-mm^2^ ellipse ROI was placed in the scala tympani in the posterolateral part of the basal turn of the cochlea on both sides. **c** Axial 3D TSE FLAIR image at the level of the floor of the 4th ventricle. A 30-mm^2^ circular ROI was placed in the pons. **d** Axial 3D SPACE FLAIR image at the level of the floor of the 4th ventricle. A 30-mm^2^ circular ROI was placed in the pons. The SIR was calculated as the ratio between the ROI in the basal turn of the cochlea and the pons at the level of the floor of the 4th ventricle

Quantitative assessment or measurement of the PE was performed by placing a region of interest (ROI). A 3-mm^2^ elliptical ROI was placed in the scala tympani of the basal turn of the cochlea and a 30-mm^2^ circular ROI was placed in the pons at the level of the floor of the fourth ventricle **(**Fig. 1**)**. The scala tympani of the basal turn of the cochlea is chosen as its volume is stable regardless of the degree of endolymphatic hydrops and can be considered as one of the largest areas in the membranous labyrinth. The brainstem at the level of the pons was chosen as reference because of its large and homogeneous aspect, lack of clear enhancement, and absence of any artifact running through it. The SIR—which can be regarded an indirect evaluation of SNR—was defined as the signal intensity of the basal turn of the cochlea divided by that of the pons. The mean SIR value was calculated for each ear.

### Statistical analysis

With SPSSv27, we performed logistic regression to identify the appropriate variables and calculate their sensitivities and specificities for denoting MD versus non-MD. Kappa analysis was done to investigate the agreement between the readers as well as to assess the test–retest reliability. To compare the average SIR values, we used paired *t*-test. We used logistic regression to calculate the specificity and sensitivity of the different MRI measures.

## Results

Twenty-nine patients (58 ears) with unilateral definite MD were selected for this study. Sex distribution was 13 (45%) females and 16 (55%) males. The median age of the female definite MD patients was 59 years (SD 9.1) and was 58.6 years (SD 12.3) for the male definite MD patients.

The time in months between the first MD attack and the MRI scan for the whole group was on average 58 with a SD of 67 months. The median was 34.4 months and median absolute deviation (MAD) 18.7 months. The time between last MD attack and the MRI scan was on average 4.3 months with SD 8.6 months. The median was 1.4 months with MAD 1.1 month.

### Endolymphatic hydrops

The distribution of patients throughout the different stages of cochlear EH on 3D SPACE FLAIR was 3 patients with grade 0 (10.3%), 9 patients with grade 1 (31%), and 17 patients with grade 2 (58.6%).

The distribution of patients throughout the different stages of vestibular EH on 3D SPACE FLAIR was 4 patients with grade 0 (13.8%), 4 patients with grade 1 (13.8%), 7 patients with grade 2 (24.1%), and 14 patients with grade 3 (48.3%).

In 4 cases, EH was found in the contralateral clinically normal ear of which 2 cases of cochlear EH grade 1 and 2 other cases of vestibular EH grade 1.

Kappa values evaluating intra-observer agreement for cochlear hydrops grading for both readers and both ears were 0.63 (substantial) for 3D TSE FLAIR and 0.87 (excellent) for 3D SPACE FLAIR. For vestibular hydrops grading, kappa values were 0.65 (substantial) for 3D TSE FLAIR and 0.80 (substantial) for 3D SPACE FLAIR. Kappa values evaluating inter-observer agreement for cochlear hydrops grading were 0.57 (moderate) for 3D TSE FLAIR and 0.87 (excellent) for 3D SPACE FLAIR. For vestibular hydrops grading, kappa values were 0.63 (substantial) for 3D TSE FLAIR and 0.76 (substantial) for 3D SPACE FLAIR (Table 2).

**Table 2:** Intra- and inter-observer reliability in 3D TSE FLAIR and 3D SPACE FLAIR of EH and PE in definite MD (both readers, both ears)

	3D TSE FLAIR		3D SPACE FLAIR	
	Intra-observer reliability	Inter-observer reliability	Intra-observer reliability	Inter-observer reliability
Cochlear EH grading	κ 0.63	κ 0.57	κ 0.87	κ 0.87
Vestibular EH grading	κ 0.65	κ 0.63	κ 0.80	κ 0.76
PE visual	κ 0.70	κ 0.57	κ 0.86	κ 0.78
PE measured	κ 0.97	21% discrepancy	κ 0.94	18% discrepancy

**Fig. 2:**
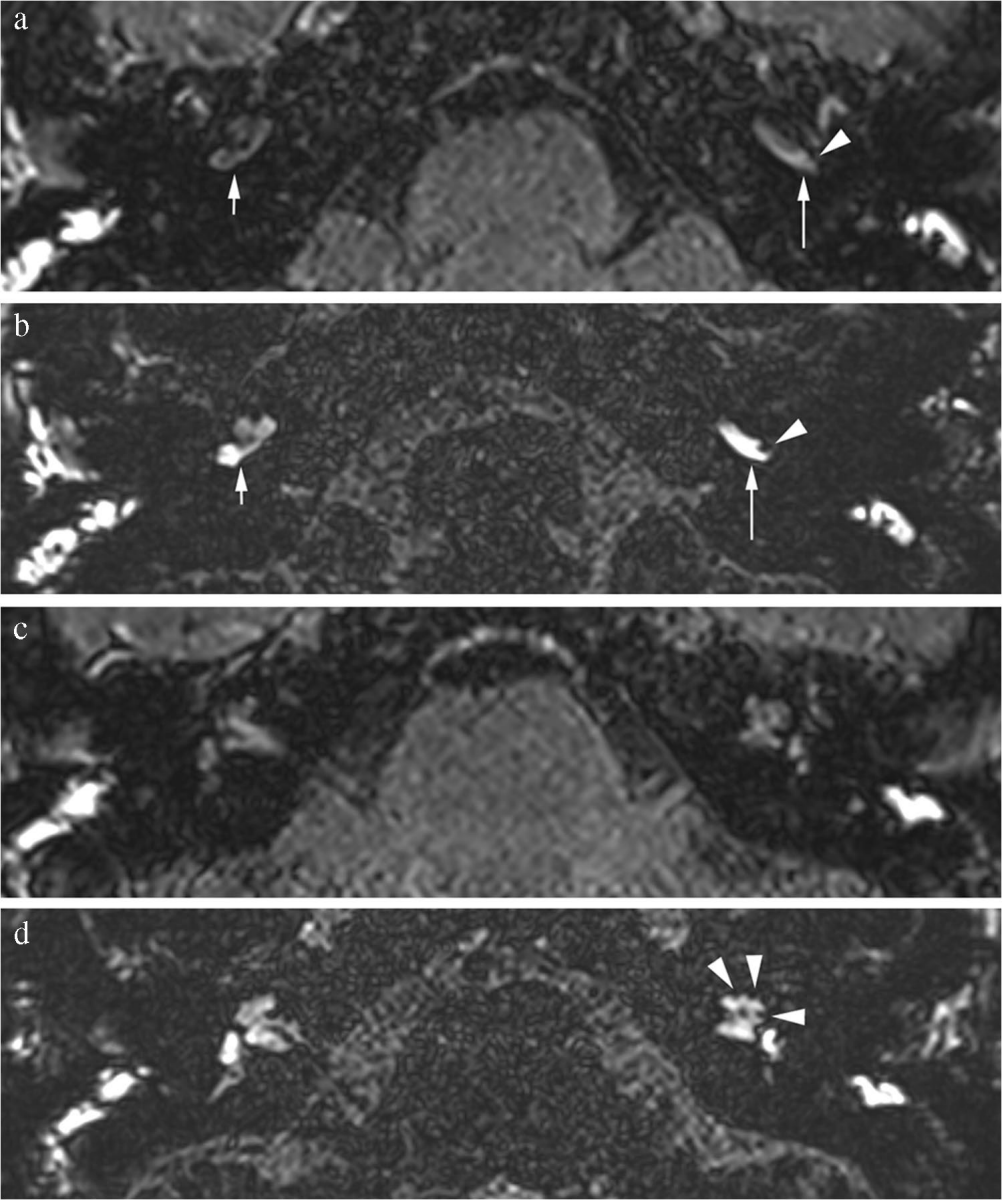
Forty-two-year-old female with a recent history of vertigo attacks associated with hearing loss and pressure sensation in the left ear. Audiometry reveals low-frequency sensory-neural hearing loss on the left side. Clinically, patient was classified as definite MD. Imaging performed 4 h after IV administration of a single dose of Gd. **a** Axial 3D TSE FLAIR image at the level of the basal turn of the cochlea. There is an asymmetrical perilymphatic enhancement, more pronounced on the left (large arrow) than on the right (small arrow). **b** Axial 3D SPACE FLAIR image at the level of the basal turn of the cochlea. There is an asymmetrical perilymphatic enhancement, more pronounced on the left (large arrow) than on the right (small arrow), which is also more clearly visible than on the 3D TSE FLAIR sequence. Note the slightly dilated endolymphatic space protruding as a small black cut-out (arrowhead) into the posterolateral aspect of the basal turn of the cochlea compatible with a grade 1 cochlear EH, which is not visible on the 3D TSE FLAIR sequence. Compare to Fig. 2a. **c** Axial 3D TSE FLAIR image at the level of the mid and apical turn of the cochlea shows no clear abnormalities. **d** Axial 3D SPACE FLAIR image at the level of the mid and apical turn of the cochlea showing the dilated endolymphatic space appearing as small black cut-outs (arrowheads) in the periphery of the mid and apical turn of the cochlea, compatible with a grade 1 cochlear EH. These abnormalities cannot be seen on the axial 3D TSE FLAIR sequence. Compare to Fig. 2c. A grade 1 vestibular hydrops in this patient was also seen (not shown)

**Fig. 3:**
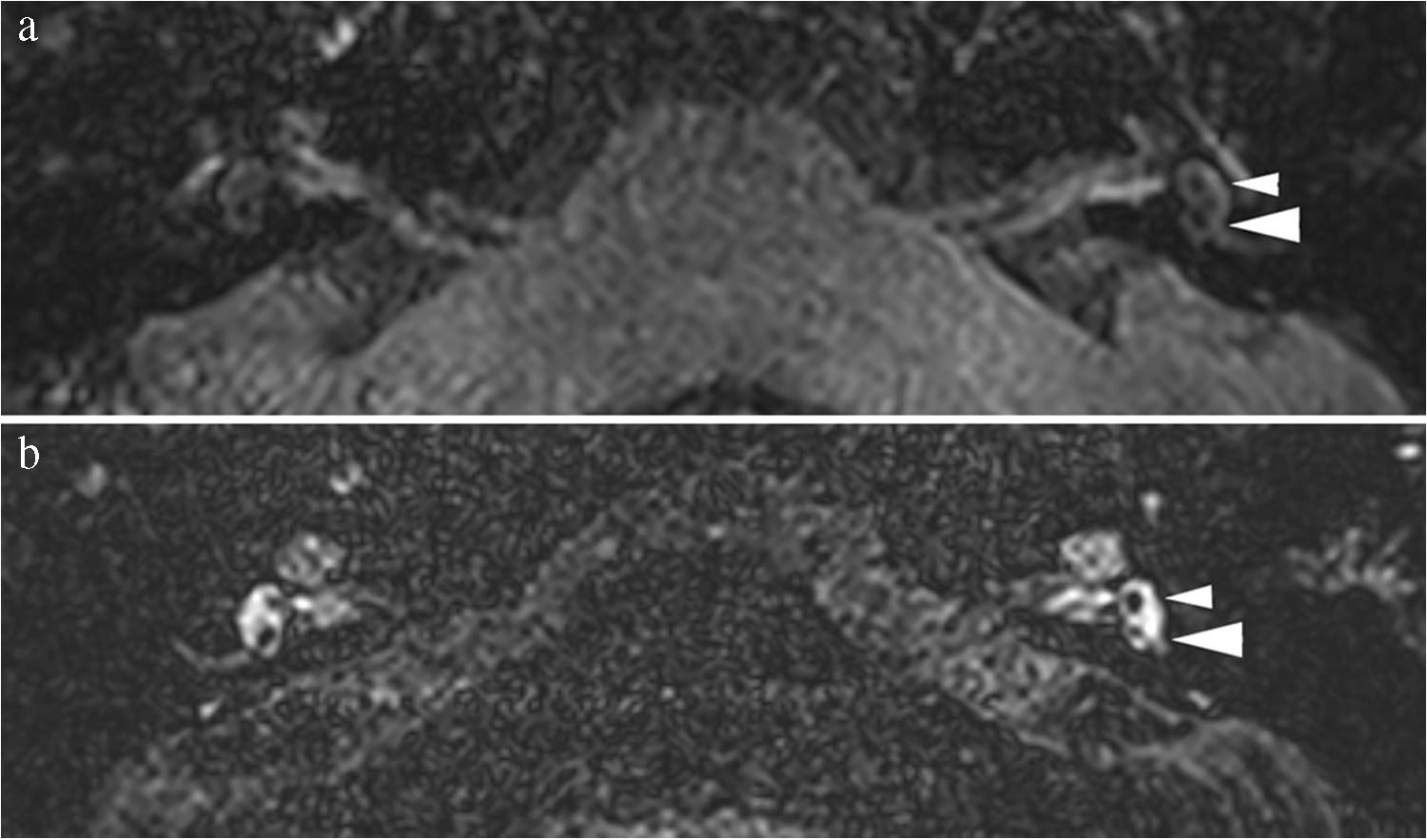
Sixty-one-year-old male with attacks of vertigo, left-sided hearing loss associated with tinnitus and pressure sensation, clinically categorized as definite MD. Imaging performed 4 h after IV administration of a single dose of Gd. **a** Axial 3D TSE FLAIR image at the level of the vestibule. Normal aspect of the saccule (small arrowhead) and utricle (large arrowhead) on the left side. Difficult visualization of the saccule on the right side. No signs of vestibular hydrops. **b** Axial 3D SPACE FLAIR at the level of the vestibule. No abnormalities on the right side, with a saccule appearing smaller than the utricle. On the left side, size relation between saccule and utricle is inverted with a saccule (small arrowhead) being larger that the utricle (large arrowhead) without being confluent: vestibular EH grade I on the left side. Note the much higher signal intensity and sharper delineation of the perilymphatic and enlarged endolymphatic spaces on the 3D SPACE FLAIR compared to the 3D TSE FLAIR, both at the level of the cochlea and at the level of the vestibule. No cochlear hydrops was seen in this patient (not shown)

The sensitivity for 3D TSE FLAIR to detect cochlear hydrops in definite Menière patients was 0.78 with a specificity of 1.00, and for 3D SPACE FLAIR sensitivity was 0.86 with a specificity of 0.88. For vestibular hydrops, sensitivity for 3D TSE FLAIR was 0.80 with a specificity of 0.95, whereas for 3D SPACE FLAIR, sensitivity was 0.85 with a specificity of 0.86 **(**Table 3)** (**Figs. 2, 3**)**.

**Table 3:** Specificity and sensitivity in 3D TSE FLAIR and 3D SPACE FLAIR of EH and PE in definite MD (both radiologist first readings, *n* = 58)

	3D TSE FLAIR	3D SPACE FLAIR
Cochlear EH	Spe 1.00	Spe 0.88
	Sen 0.78	Sen 0.86
Vestibular EH	Spe 0.95	Spe 0.86
	Sen 0.80	Sen 0.85
PE visual	Spe 0.64	Spe 0.71
	Sen 0.98	Sen 1.00
PE measured	Spe 0.72	Spe 0.72
	Sen 0.60	Sen 0.62
EH + PE visual	Spe 0.98	Spe 0.98
	Sen 0.85	Sen 0.91
EH + PE measured	Spe 0.98	Spe 0.93
	Sen 0.83	Sen 0.86

### Cochlear blood perilymph barrier impairment

#### Qualitative analysis

Kappa values evaluating intra-observer agreement for visual comparison of PE were 0.70 (substantial) for 3D TSE FLAIR and 0.86 (excellent) for 3D SPACE FLAIR. For inter-observer agreement, kappa values were 0.57 (moderate) for 3D TSE FLAIR and 0.78 (substantial) for 3D SPACE FLAIR **(**Table 2**)**.

The sensitivity for 3D TSE FLAIR for the visual PE assessment was 0.98 with a specificity of 0.64. For 3D SPACE FLAIR, sensitivity is 1.00 with a specificity of 0.71 **(**Table 3**)**.

#### Quantitative analysis

Kappa values evaluating intra-observer agreement for measured ratio analysis of PE of the basal turn of the cochlea to the brainstem were 0.97 (excellent) for 3D TSE FLAIR and 0.94 (excellent) for 3D SPACE FLAIR. Inter-observer agreement for measured ratio analysis of PE showed a 21% discrepancy for 3D TSE FLAIR and an 18% discrepancy for 3D SPACE FLAIR **(**Table 2**)**.

The sensitivity for 3D TSE FLAIR for the measured BLB impairment alone was 0.60 with a specificity of 0.72. For 3D SPACE FLAIR, sensitivity was 0.62 with a specificity of 0.72 **(**Table 3**)**.

For the MD ears, the SIR was significantly lower (*p* < 0.001) for 3D TSE FLAIR (mean 161.28; SD 88.59) than for 3D SPACE FLAIR (mean 1212.07; SD 507.54). For the control ears, there were also significant (*p* < 0.001) differences in the SIR between 3D TSE FLAIR (mean 114.54; SD 31.83) and 3D SPACE FLAIR (mean 887.8; SD 236.06). There was also a significant difference in SIR between MD ears and control ears for 3D TSE FLAIR as well as for 3D SPACE FLAIR **(**Table 4**)**.

**Table 4:** SIR between PE in the scala tympani of the basal turn of the cochlea and the pons at the level of the floor of the fourth ventricle in 3D TSE FLAIR and 3D SPACE FLAIR for both, MD ears (*n* = 29) and control ears (*n* = 29)

	SIR MD ears Mean	Standard deviation	SIR control ears Mean	Standard deviation	*p*-value
3D TSE FLAIR	162	89	115	32	< 0.001
3D SPACE FLAIR	1212	508	888	236	< 0.001

### Diagnostic accuracy

The sensitivity for the combination of EH and visual PE was 0.85 with a specificity of 0.98 for 3D TSE FLAIR. For 3D SPACE FLAIR, sensitivity was 0.91 with a specificity of 0.98. The sensitivity for the combination of endolymphatic hydrops and measured PE was 0.83 with a specificity of 0.93 for 3D TSE FLAIR. For 3D SPACE FLAIR, sensitivity was 0.86 with a specificity of 0.93 **(**Table 3**)**.

## Discussion

The principle of hydrops imaging on MRI is based on the fact that Gd penetrates through the blood-perilymph barrier but not through the blood-endolymph barrier thus enhancing the perilymphatic space in which the dilated non-enhancing endolymphatic spaces become visible in case of hydrops. Two types of Gd administration are used. In the IT technique, a diluted Gd contrast solution is injected—after local anesthesia—in the middle ear and is subsequently resorbed by the oval and round window. Imaging is performed after 24 h. For the IT technique, 3D real IR sequences are most frequently used. At the onset, a single sequence was used in which—by setting the inversion time between the null point of Gd-containing perilymph and that of the endolymph fluid without Gd—endolymphatic space, perilymphatic space, and surrounding bone can be separately visualized. Later on, image series with different inversion times were subtracted from each other with also subtraction of MRC images [7–10]. However, this process is time-consuming and carries the risk of misregistration [2, 4, 6].

The IV technique has the advantage that it is less invasive, not an off-label use of Gd, and that both ears can be imaged at the same time. Yet, concentration of contrast in the perilymphatic space is lower in the IV technique than in the IT technique. Therefore, the use of double and even triple dose of contrast was advocated in the beginning to overcome this lower concentration of contrast. However, with the introduction of optimized sequences, single-dose Gd administration is now considered sufficient [1, 2, 4, 6]. The maximum level of contrast is reached between 3.5 and 4.5 h after IV administration [14, 15]. Thin slice isotropic 3D TSE FLAIR is currently the most frequently applied IV post-Gd EH imaging sequence [1–6]. It is a robust sequence, requiring no postprocessing, with a very high diagnostic accuracy for the detection of EH and asymmetrical PE in patient with definite MD [2–6]. Whereas in the beginning non-isotropic sequences were used, nearly all sequences currently applied in literature are isotropic [1, 3]. The combination of EH and asymmetrical PE has been reported as highly sensitive and specific for definite MD [3], allowing even the differential diagnosis with vestibular migraine and other vertigo-associated inner ear pathologies [5]. hT_2_w-3D-FLAIR sequences have been reported to be sensitive to very low concentration of Gd [11, 12] with a better image quality [16]. Higher repetition times have hereby also been reported to increase SI [17]. In this study, we demonstrate that 3D SPACE FLAIR with CFA is superior to conventional 3D TSE FLAIR regarding SNR, the evaluation of BLB impairment, and cochlear and vestibular EH with higher reliability in patients with definite MD.

### Endolymphatic hydrops detection

3D SPACE FLAIR proves to have a higher intra- and inter-observer reliability for the detection of cochlear and vestibular EH compared to 3D TSE FLAIR, meaning that 3D SPACE FLAIR is a more robust and reproducible sequence, compared to 3D TSE FLAIR in the detection of EH, in patients with definite MD.

The higher sensitivity of 3D SPACE FLAIR for cochlear and vestibular EH alone is explained by the higher SIR of the 3D SPACE FLAIR resulting in a much better and sharper delineation of both perilymphatic and endolymphatic spaces in the membranous labyrinth, especially in the lower grades of cochlear and vestibular hydrops **(**Fig. 2 and 3**)** [11]. In this study, the number of high-grade cochlear and vestibular EH outranges the number of lower grades. Probably sensitivity could have been even higher if a larger number of low grades EH cases would have been included in the study as lower grades of EH are believed to benefit more from the increased SNR in 3D SPACE FLAIR.

We have also found 4 cases of clinically silent EH—2 cochlear hydrops grade 1 as well as 2 vestibular hydrops grade 1—in the contralateral normal ear. This is in line with previous reports in literature [1–6] and can be explained by the fact that MD is considered a continuous spectrum of disease in which the clinical image as well as the disease stage aggravates over years with very frequently involvement over time of the contralateral ear.

### Cochlear blood perilymph barrier impairment

#### Qualitative evaluation

3D SPACE FLAIR has a significantly higher intra- and inter-observer variability for the qualitative or visual evaluation of PE compared to 3D TSE FLAIR meaning that 3D SPACE FLAIR is also a more robust sequence for cochlear BLB impairment.

In 3D SPACE FLAIR, the high sensitivity of 3D TSE FLAIR in the evaluation of BLB impairment alone (almost 1.0) is maintained, whereas specificity is improved from 0.64 to 0.71.

#### Quantitative analysis

In our study, quantitative or measured evaluation of PE alone has a substantial lower sensitivity and specificity in diagnosing definite MD compared to qualitative or visual evaluation for both, 3D SPACE FLAIR and 3D TSE FLAIR. This is opposed to previous reports in literature in which quantitative evaluation seems to have an added value over qualitative evaluation [5]. We believe that this difference can be explained by various factors. Quantification of MR images is still a challenging task, influenced by several variables. As compared to computed tomography images, MR images cannot be calibrated via an analog to Hounsfield units. Another factor which might explain the large standard deviation in measured evaluation is that the disruption of the BLB, which is responsible for the impaired PE, is correlated to the EH grade and duration of the disease [3, 18]. Moreover—in our experience—there seems to be a gradient in the enhancement of the basal turn of the cochlea with a more pronounced enhancement in the posterolateral part of the basal turn. A ROI placement and measurement is performed on a single position on a single slice through the basal turn of the cochlea whereas visual evaluation is performed by scrolling through all the images of the entire cochlea. Besides patient-related factors and tissue parameters, parameters such as electronic signal amplification also might influence measured values.

SIR proves to be significantly lower in the 3D TSE FLAIR sequence than in the 3D SPACE FLAIR in the MD ears as well as for control ears. This is in line with the prior findings of Nahami et al. and Naganawa et al., proving the higher SIR of the 3D SPACE FLAIR with CFA [11, 14]. In addition, there is a significant difference in SIR between the MD ears and the control ears, with both 3D SPACE FLAIR and 3D TSE FLAIR. This is in contradiction with the paper of Nahami et al. [11], but confirms the findings of Bernaerts et al., in which asymmetrical PE has been reported in definite MD ears and can be regarded as a very sensitive and specific sign of definite MD [3, 5].

Combined grading of EH with qualitative or visual evaluation of PE results in the highest sensitivity and specificity for detection of definite MD for both sequences. This is also in line with prior findings in which the combination of EH and PE yielded the highest sensitivity and specificity for definite MD [3, 5]. In the current study, however, this combined approach has an even higher diagnostic accuracy in 3D SPACE FLAIR, with a gain of 6% in sensitivity and equal high specificity compared to the already high sensitivity and specificity of 3D TSE FLAIR.

### Limitations of this study

One of the limitations of this study is that the contralateral clinically normal ear of the definite MD patients was used as a control group and that these control ears also contained 4 cases of clinically silent low-grade EH. Apart from the fact that in a retrospective study it is almost impossible to form such a matched control group, it is also for ethical reasons not allowed to inject a group of normal healthy volunteers with Gd to see the hydrops features in a normal population. Since the clinical, auditory, and vestibular functional tests for MD—based on the 2015 Bárány Society criteria—are still considered gold standard for the diagnosis of MD, and as in the majority of cases MD clinically can be confined to one ear, we could use the healthy contralateral normal ears as the control group.

We did not use the remaining of the 85 scanned ears with other pathologies as control ears because then the control group would outnumber significantly the MD group with impact on the sensitivity and specificity and with an increased variability in particular for the SIR. Moreover, these patients showed a very heterogeneous distribution of pathologies in which it is often yet uncertain what a delayed Gd-protocol would yield.

### Conclusion

In summary, the 3D SPACE FLAIR sequence with CFA has a higher reproducibility and reliability than 3D TSE FLAIR for the visualization of EH and increased PE with a single-dose IV administration of Gd in patients with definite MD. The combination of EH and visual PE evaluation has the highest diagnostic accuracy for detection of definite MD on 3D SPACE FLAIR with a raise in sensitivity of 6% and equal high specificity compared to 3D TSE FLAIR. The increased TR and TE give much more heavily T2-weighted images, which benefits SNR. Furthermore, the increased echo train length significantly reduces acquisition time, which favors patient comfort and minimizes motion artefacts. In our hospital, we therefore replaced 3D TSE FLAIR by 3D SPACE FLAIR resulting also in a significant reduction of the scan time of almost 50%.

## Data Availability

All data and materials support our published claims and comply with field standards.

## Abbreviations

EH: Endolymphatic hydrops
MD: Menière’s disease
GD: Gadolinium
MRI: Magnetic resonance imaging
PE: Perilymphatic enhancement
IV: Intravenous
IT: Intratympanic
3D: Three-dimensional
TSE: Turbo spin-echo
FLAIR: Fluid-attenuated inversion recovery
IR: Inversion recovery
MRC: Magnetic resonance cisternography
hT_2_w-3D-FLAIR: Heavily T2-weighted 3D FLAIR
CFA: Constant flip angle
SPACE: Sampling Perfection with Application optimized Contrasts using different flip angle Evolution
RF: Radiofrequency
VFA: Variable flip angle
TE: Echo time
SIR: Signal intensity ratio
TR: Repetition time
SNR: Signal-to-noise ratio
BLB: Blood-labyrinth barrier
ROI: Region of interest
MAD: Median absolute deviation

## Code availability

All software application and custom code support our published claims and comply with field standards.
